# Supplementary material for: CGGBP1-regulated cytosine methylation at CTCF-binding motifs resists stochasticity
Source: BMC Genet. 2020 Jul 29;21:84. doi: 10.1186/s12863-020-00894-8 (PMC7392725; doi:10.1186/s12863-020-00894-8)
Supplement: Supplementary file 1 — Additional file 1. Primer names, locations, sequences and annealing temperatures used for the candidate region methylation analyses shown in Additional file 14. The cyclic denaturation (95 °C, 30 s) and extension (70 °C) steps were the same for all primer combinations. Data was collected post-extension at 80 °C as mentioned in methods. [file 12863_2020_894_MOESM1_ESM.pdf]

TEMPLATE	LEFT PRIMER	RIGHT PRIMER	ANNEALING TEMPERATURE
Alu	GAGGCTGAGGCAGGAGAATCG	CGCCCAGGCTGGAGTGCAGTGGCGCG	55
CPG-1	CGAGAGCACTACGCAGTCAG	TCTGACACCTAAGCCCTACCA	53.5
CPG-11	ATTGCCTCACCTGGGAAG	GAGATTCCGTGGGCGTAG	54.5
CPG-10	CGGGCCAGTGACAAAGAG	GCCATGGAGTCCTACGATGT	54.5
CPG-16	TGGGACCTAGAGAACCGAGA	ATTGAGACATCAGCGGCATT	54
CPG-5	GTGCCCAGGTAGAAGCAGAG	CGTCTCCATGTGCTGCTTT	54
CPG-7	AGAACAGCGATTCCTTCGAG	AGTCCCTCGGCCAGTTTATC	54
CTCF-1-1	TGCATCTGCAGAGAAGGAGA	AGCGAGACATACGCAGACCT	55
CTCF-1-2	TGGTACTGCACCACTCTGGA	GAAAGTCCTAGCGGATCTGG	55
CTCF-16	AGGTGCAGGGAATAATGCAG	TGCTTCCAGACATGGGTATG	54
CTCF-2-2	CACACGTTTGGTGGCTTAAA	AAGGCCGGGTAAAGACAGAG	53
CTCF-2-2	GGCGTCAGTCAAGTGATGG	TGTGAGGCGATTTAAACGTG	53
CTCF-3-1	TGTTCATTTTTCAAAGTGAGCTTTA	ATGTGTGCGTCTGTTTCTGC	53
CTCF-9-1	GCCATCTAGTGGTGCTGTTG	GTGAGCAATGTTCCCACCTT	55
CTCF-9-2	GCCACCAGATGGCACTATTT	ACCGAATTGCCTCAGAACAG	55
CTCF-CHRX	TGCAGGAAATTCATGAGCTG	CATCCCTGGAACACAGATCC	53
FAM50B	GTGGTTCTCGTGGAGGTCAG	GCACCAATTTCCAGCATTTT	52
GRB10	CGAAAGCCCTCCATGTCTAC	CCTTCCTGGTTCTTGCTCTG	55
IGF2	CCTGCCTAGAGCTCCCTCTT	TTTCATATTCCGTGCCATGA	52
L3MBTL	GTTTCTGCCACCGTCCTG	CTGCGGTTTACGAGGCTTAG	55
MEG3-1	CCAACGTCCCACGTCTCTAT	AGGGCTGAAAAGAAGGGAGA	55
MEG3-2	GACTTTGGAGGCGTGATGTT	CCTGCTACAGGGAAAAGCAG	55
MEST	GTCCAGCACCGAACCTTTC	TTAAAAGGCATCCCTTACGC	54
NAP1L5	TTAGAGGAGAAGCCGCAGAG	GCCCAGAAGATGTCAGGGTA	55
NDN	AAGGTGGAGTGCTTCTTCCA	GAGTTTGCGCTGGTCAAAG	55
NNAT	TTCTGGAAGGGGGCTAAGAT	TGGTCGAGAAAGAGGGTTTG	56
PEG10-1	AGTCCTCGCGTGGTGAGTAT	GTTTTGGGGTACAGCCAGTG	54
PEG10-2	CGCCTCCTCTTAGGTAGCC	CTGAAGGGAGGGGCTGAG	54
PLAGL1	GGAGGACCTAAGCTGGGTGT	ATCGGGACAGCTGATAATGC	55
RB1	GCGAGCAGCTCTCACCTCT	CCACGCTTCTACTCCCTGAA	55
RB-L1-1	CAGGCCAGTGTGTGTGCG	AAAGCGCAATATTCGGGTGG	55
RB-L1-2	GAGCTACGGGAGGACATTCA	CCTGAATCTGAACGTTGGCC	55
RB-L1-3	CACGCAGCTGGAGATCTG	AATACCCTGCCGTGTGAGG	55
RB-L1-4	TGCCTACAAGAGAAAGCAGGA	AGTCTTGCTAGCGGTCTATCA	55
RB-L1-5	TGAAAACCGGCACAAGACAG	GCTGAGACGATGGGGTTTTC	55
RB-L1-8	TGAAAACCGGCACAAGACAG	GCTGAGACGATGGGGTTTTC	55
SNRPN	CTTGACAATCCCCGAACACT	CACCCAGGGATGACTGACA	55
ZIM2	ATATGCCACCAACCAACCAG	CCGAGTAGGCGCTGTTCTA	55
GOM-TDG	AAGTACAAACACCAGTGACAGA	AGCATCACCTCCCATAACCT	53
GOM-DNMT1	CCTGATGCCCCTAGAACTGT	TATCTCGCCAACCTGACCTC	59
GOM-DNMT3A	ACTATAGACCAGGCGTGCTC	GCGGGTTGTGAGAAGGAATG	59
GOM-DNMT3B	TGTGTGCCCATAATGTTGCA	CCATTGTAGCCTCACCCTCA	59
LOM-TDG	CAAAGAGCTGTGATCATGCCA	GGTCATCCACTGCCCATTAG	59
LOM-DNMT1	GACCGTGGACAGCTGACT	TGAGACTGAGCCTGAATCCA	59
LOM-TET3	CATTGTGGGGTGTTGGTGG	CGCCCACCTCAGAAAACAG	59
LOM-MBD4	TTATGCTGAAAACTGGCCTTG	AAATCAGTTAACGTTTCTCCACC	59
LOM-CPG-1	TAGCTGAGCGGGAAGAAGC	GAGAACGACGGGGAGCTG	60
GOM-CPG-2	GGTCTCCGTTCCCTTCTCC	TTCGGAAGTGGAAGAAGCCT	60
LOM-CPG-3	GCTGCCTTCGAGCCTCTTC	CTAGGTGAAGAACGGGCAAC	60
