# Supplementary figures and images for: CGGBP1-regulated cytosine methylation at CTCF-binding motifs resists stochasticity
Source: BMC Genet. 2020 Jul 29;21:84. doi: 10.1186/s12863-020-00894-8 (PMC7392725; doi:10.1186/s12863-020-00894-8)

# HEK293T

---

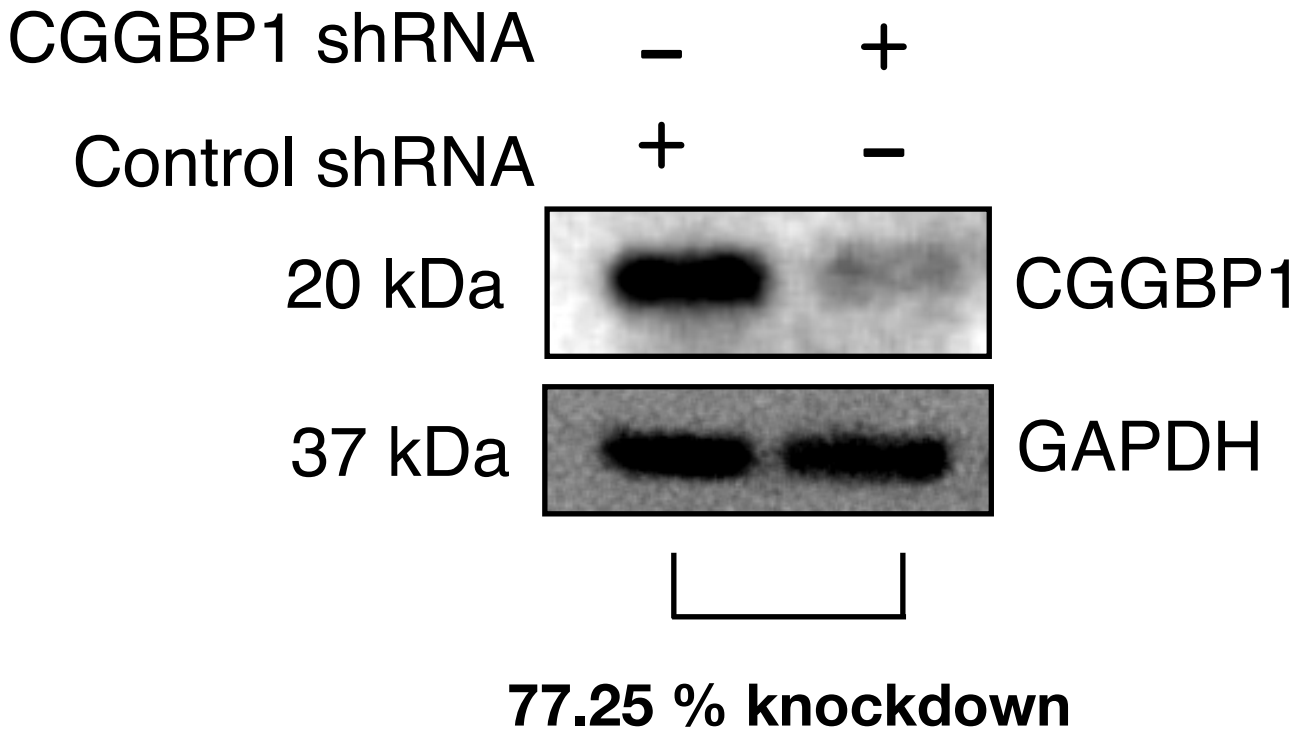

Supplement: Supplementary file 2 — Additional file 2. CGGBP1 depletion in HEK293T cells: HEK293T cells were transduced with non-targeting shRNA or CGGBP1-targeting shRNA lentiviruses. Lentivirus-transduced cells were selected with 10 μg/ml Puromycin for 1 week and subjected to immunoblotting. The level of CGGBP1 and GAPDH are shown in the upper and lower panel respectively. A CGGBP1 knockdown of approximately 77% is observed when normalized to GAPDH levels. [file 12863_2020_894_MOESM2_ESM.pdf]

# HEK293T

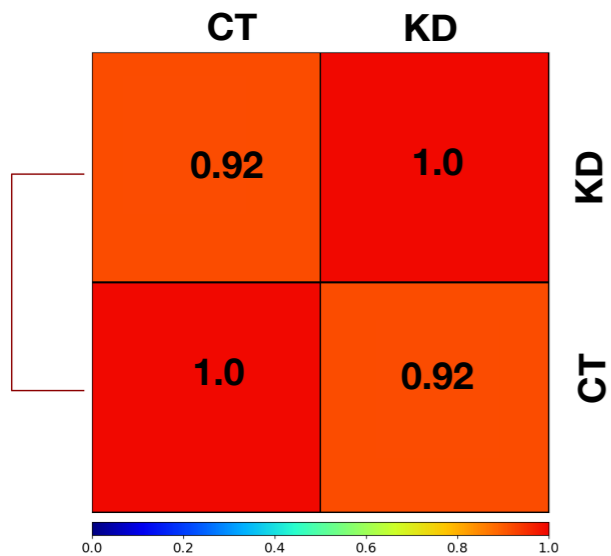

10 Kb

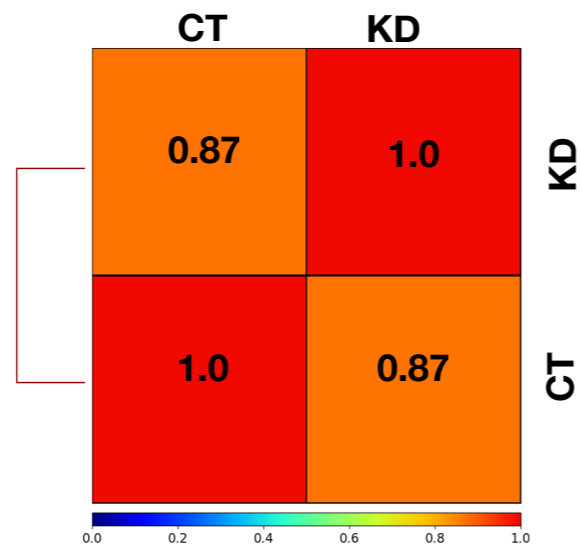

5 Kb

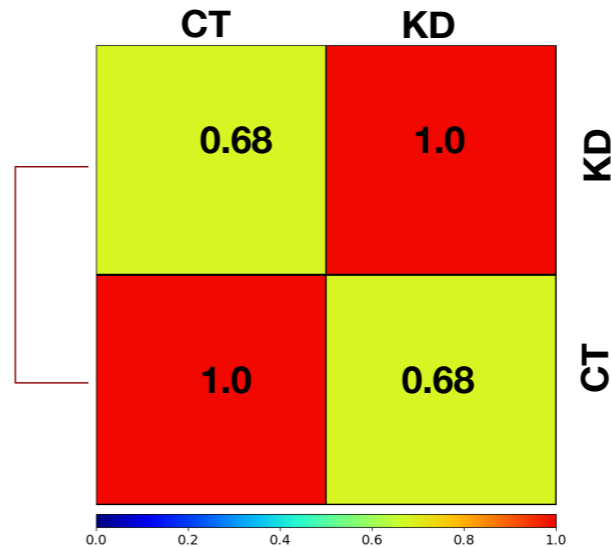

1 Kb

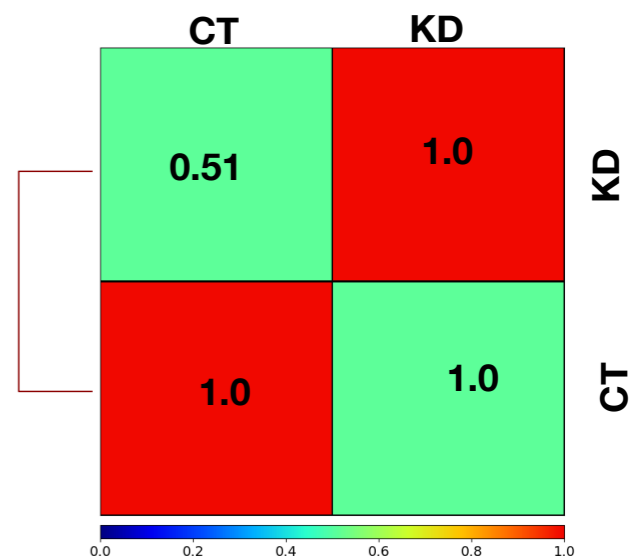

0.2 Kb

Supplement: Supplementary file 5 — Additional file 5. Methylation differences between CT and KD are discernible at small genomic length ranges. Genome-wide methylation signal distribution was compared between CT and KD by using “deeptools multiBigwigSummary”. Methylation signals were compared at bin sizes of 10 kb, 5 kb, 1 kb and 0.2 kb. Correlation between CT and KD was computed by the Spearman method by using “deeptools plotCorrelation” for HEK293T cells. [file 12863_2020_894_MOESM5_ESM.pdf]

# HEK293T

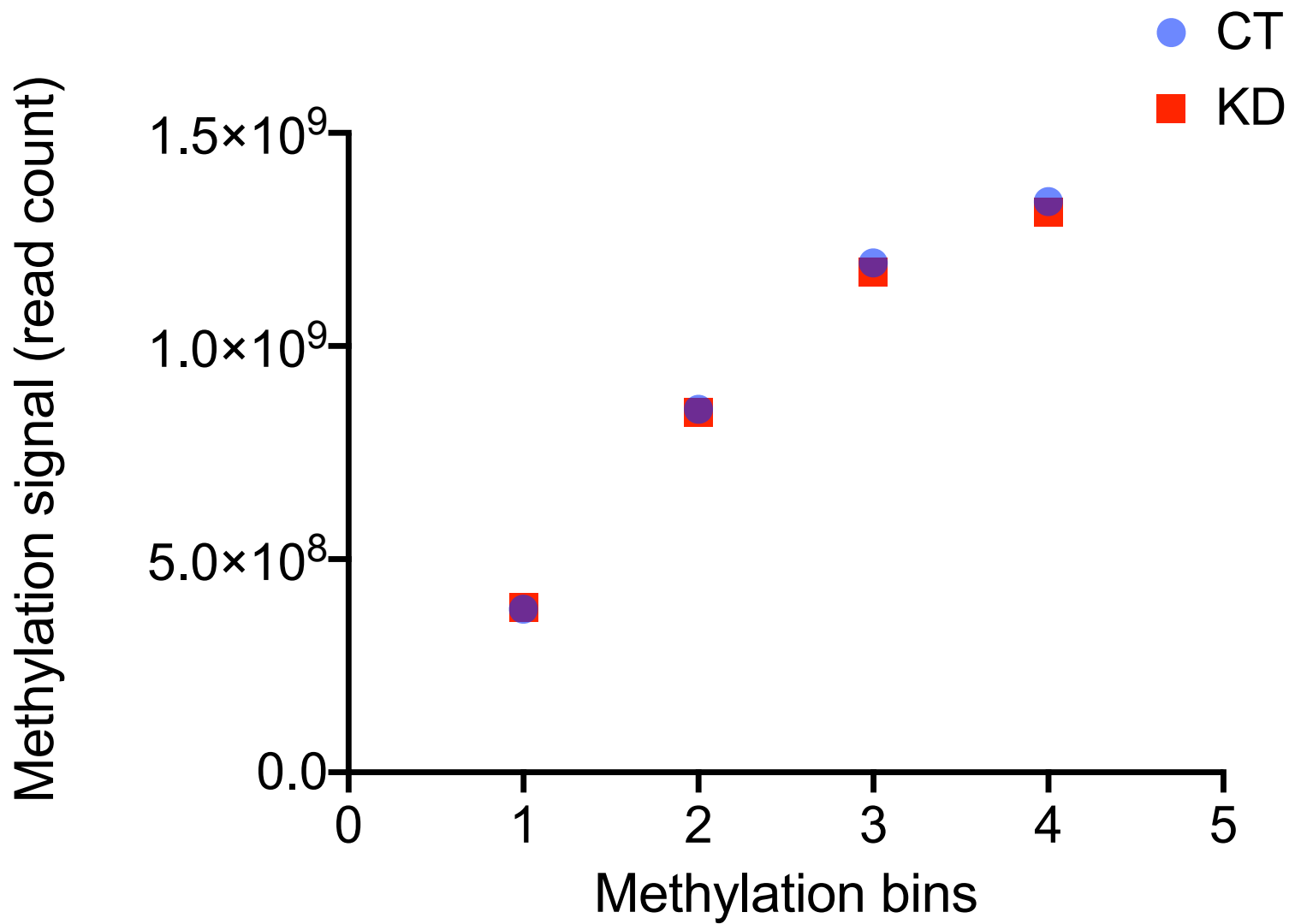

Supplement: Supplementary file 7 — Additional file 7. The MeDIP reads distribution for HEK293T CT and KD at low methylation signal bins (1 to 4): Lower methylation signal bins account for a major fraction of MeDIP reads. The frequency of the MeDIP reads for these bins with less than 5 methylation read signals were calculated separately from those in the range 5–30. [file 12863_2020_894_MOESM7_ESM.pdf]

# GM02639

---

CGGBP1 siRNA

-

+

Control siRNA

+

-

20 kDa

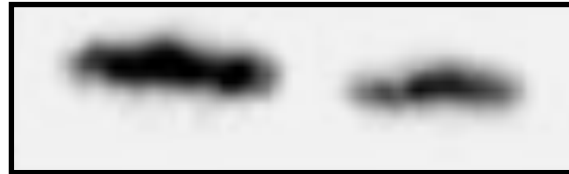

CGGBP1

37 kDa

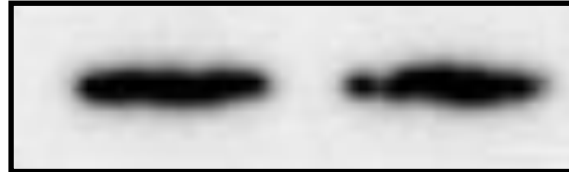

GAPDH

**55 % knockdown**

Supplement: Supplementary file 8 — Additional file 8. CGGBP1 depletion in GM02639 cells: GM02639 cells were transfected with non-targeting or CGGBP1-targeting siRNA twice at 24 and 72 h post-seeding. Cells were harvested at 96 h. Immunoblotting results for CGGBP1 (upper panel) and GAPDH (lower panel) show approximately 55% knockdown of CGGBP1 when normalized to the level of GAPDH. [file 12863_2020_894_MOESM8_ESM.pdf]

# GM02639

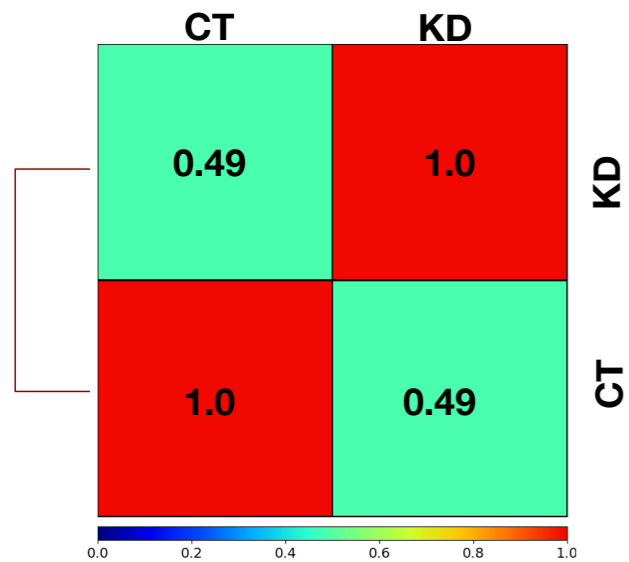

10 Kb

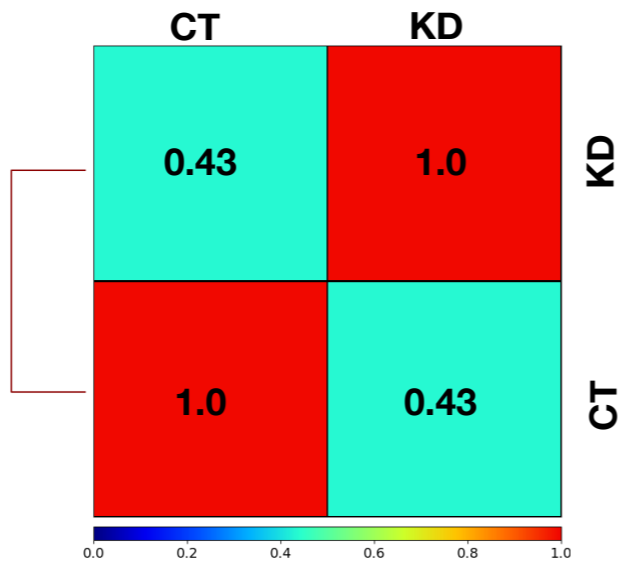

5 Kb

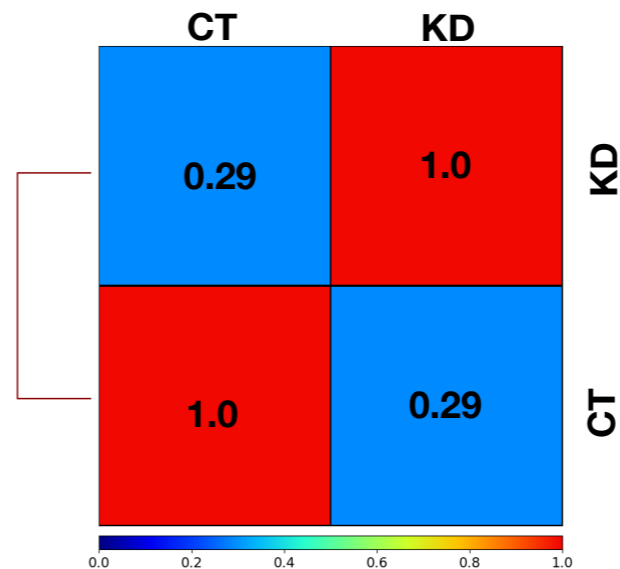

1 Kb

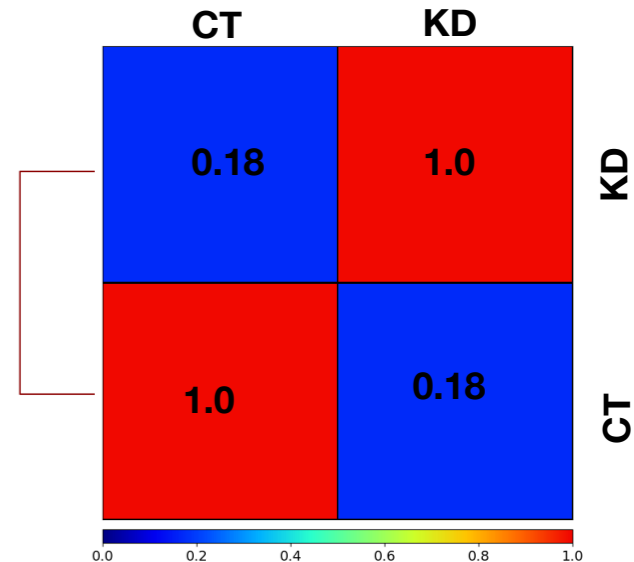

0.2 Kb

Supplement: Supplementary file 9 — Additional file 9. Methylation differences between CT and KD are discernible at small genomic length ranges: Genome-wide methylation signal distribution was compared between CT and KD by using “deeptools multiBigwigSummary”. Methylation signals were compared at bin sizes of 10 kb, 5 kb, 1 kb and 0.2 kb. Correlation between CT and KD was computed by the Spearman method by using “deeptools plotCorrelation” for GM02639 cells. These correlation coefficients can be compared with those for HEK293T (Additional file 7). [file 12863_2020_894_MOESM9_ESM.pdf]

# GM02639

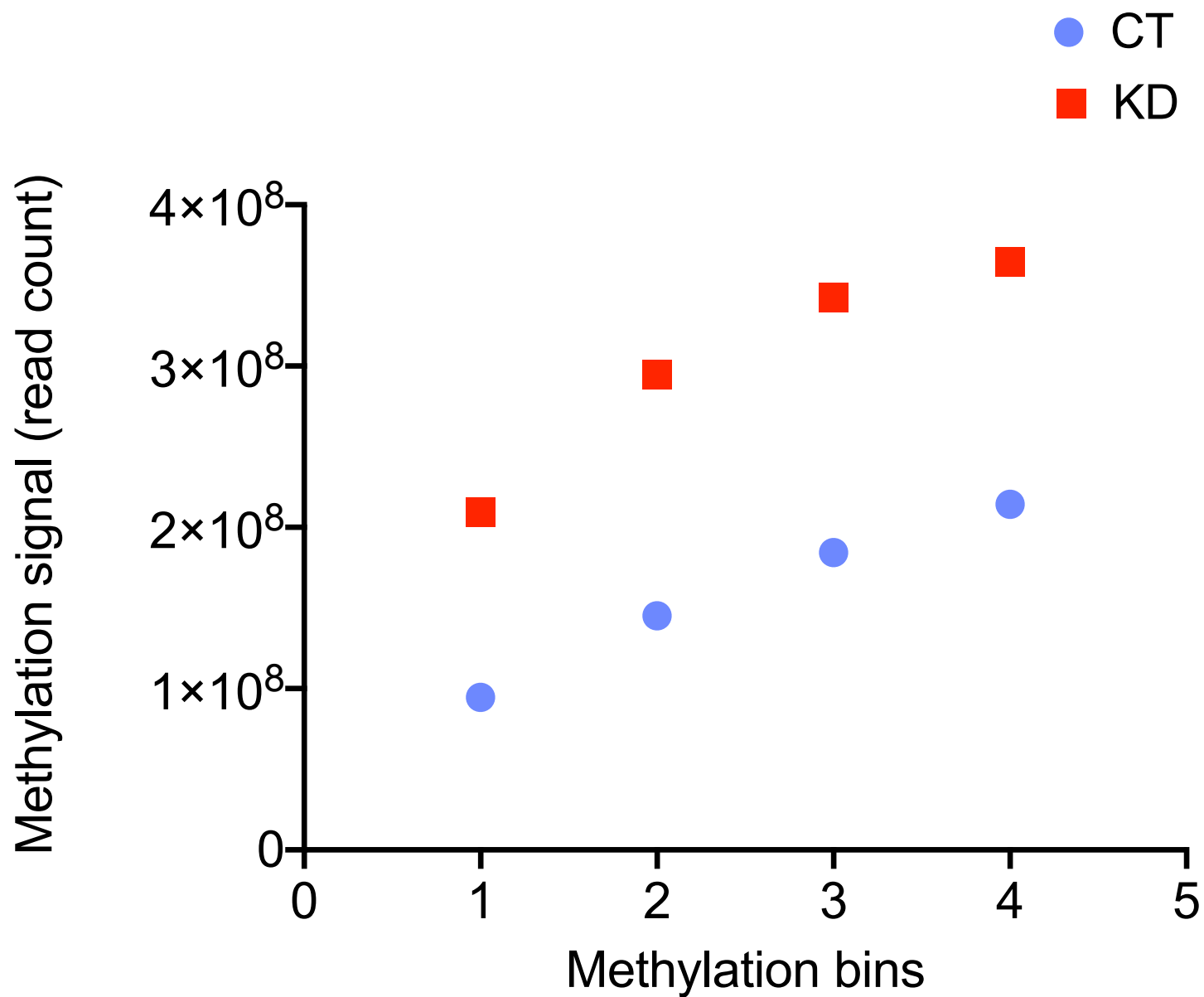

Supplement: Supplementary file 10 — Additional file 10. The figure shows the methylation reads distribution in CT and KD at lower methylation bins (1 to 4) in GM02639 cells. [file 12863_2020_894_MOESM10_ESM.pdf]

A

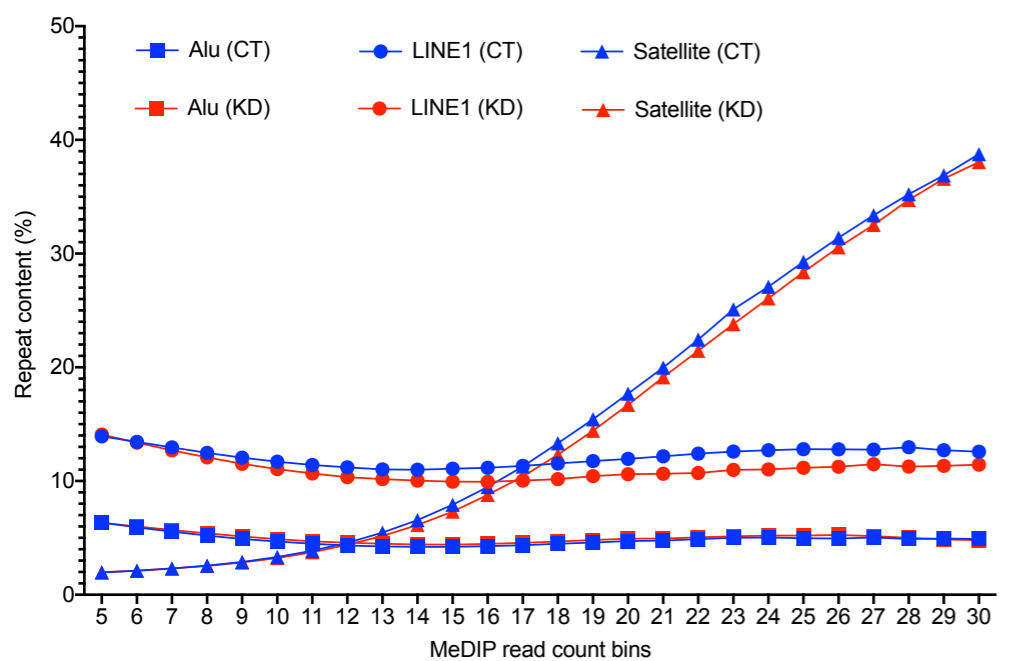

B

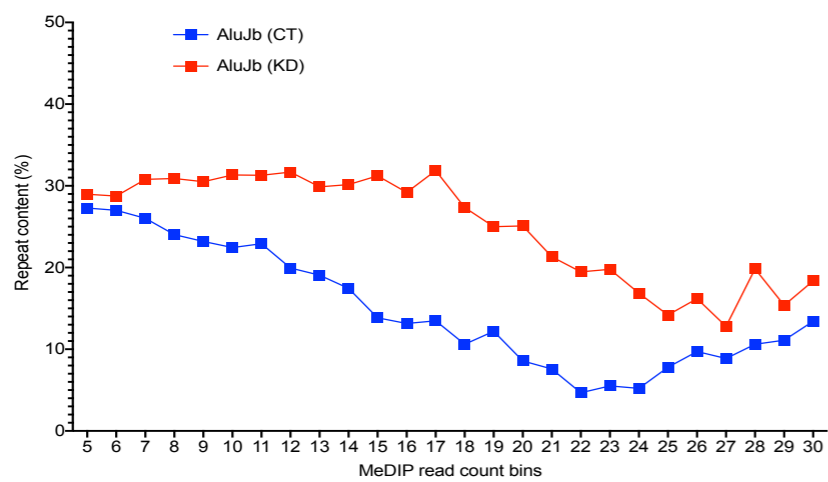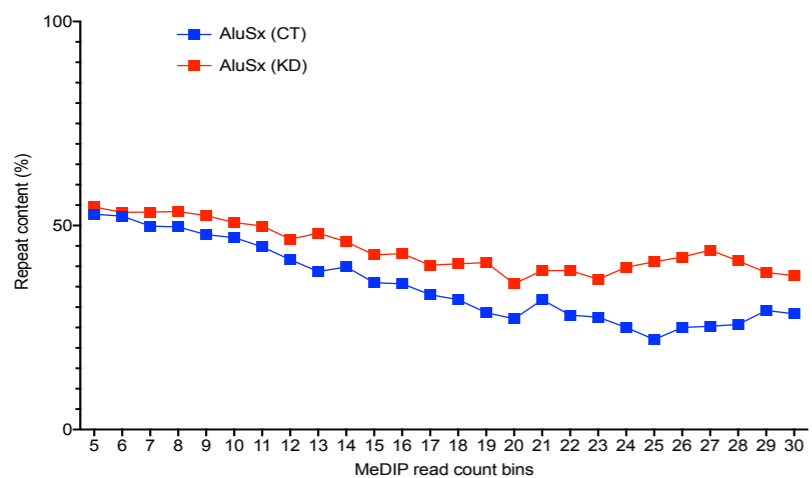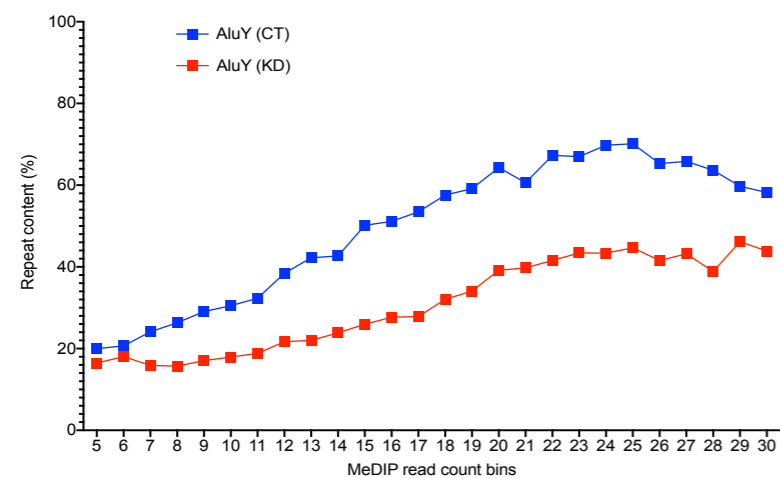

C

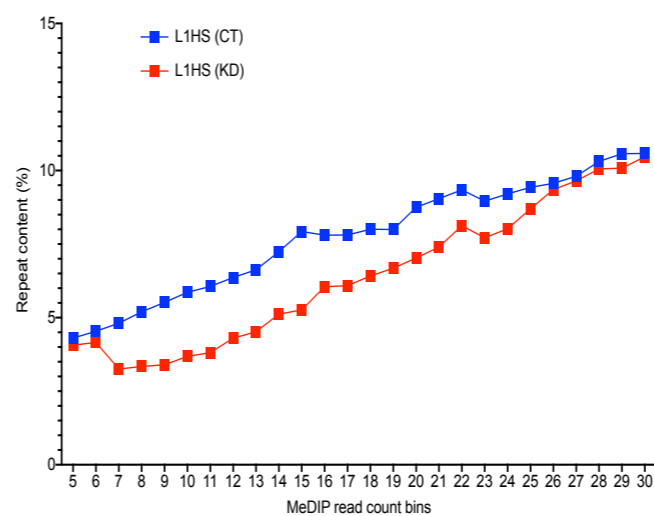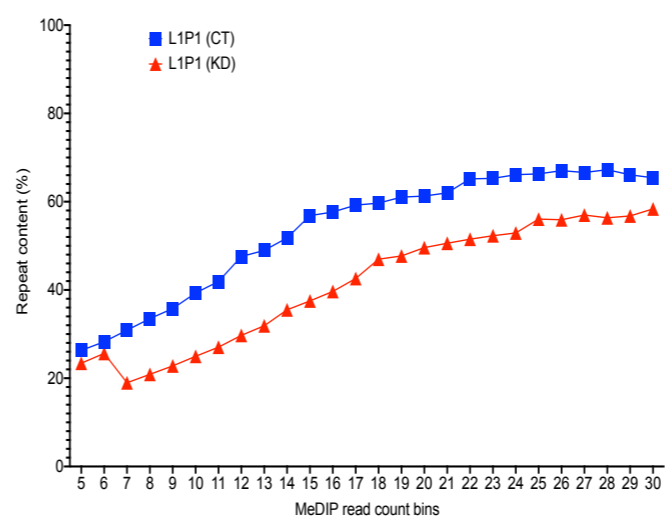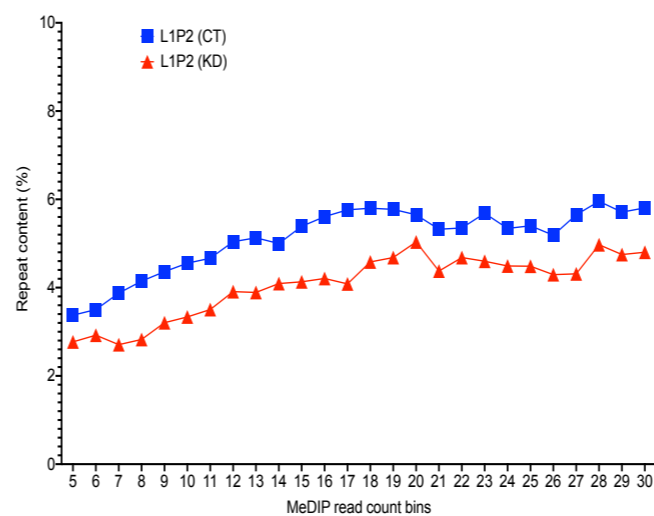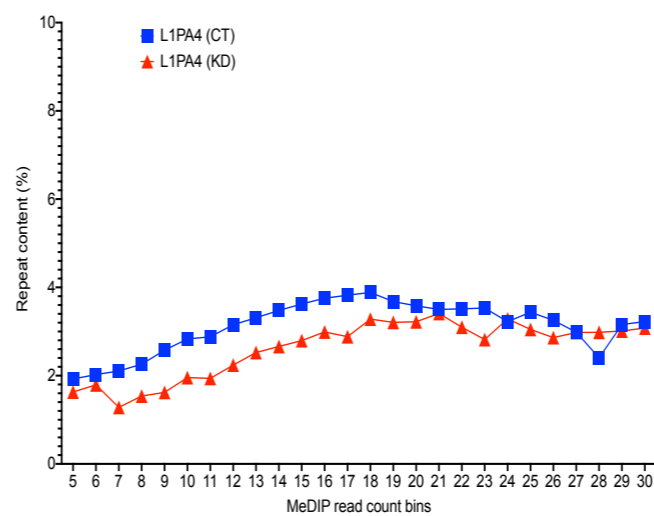

D

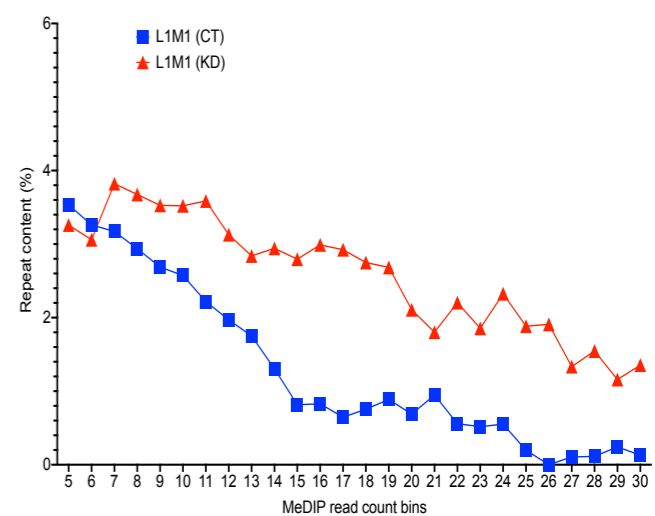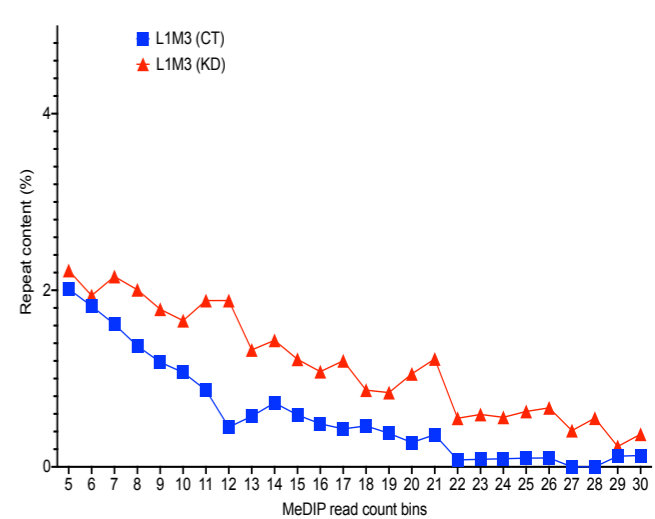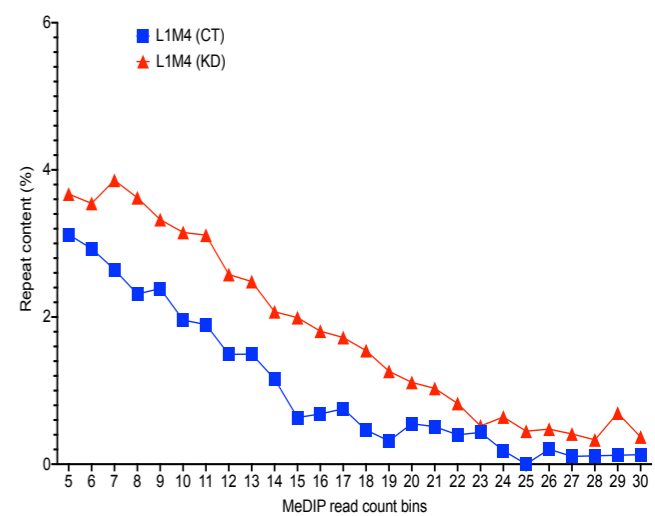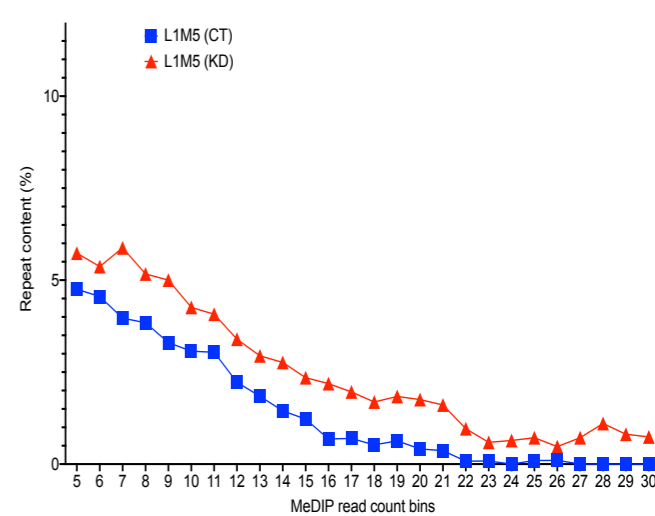

Supplement: Supplementary file 11 — Additional file 11. Repeat content analysis in HEK293T CT and KD MeDIP DNA shows subfamily-specific methylation changes: Methylation bin frequency plots for HEK293T CT and KD. CT and KD reads for each methylation bin (From 5 to 30) were merged and sequences for merged regions (> 150 bp long) were extracted and subjected to repeat identification. The figures depict the occurrence of the three most populous repeats (Satellites, L1-LINEs and Alu-SINEs). (A) No overall differences in repeat content were observed between CT and KD. (B) A classification of the Alu SINEs into J, S and Y subfamilies revealed subfamility-specific differences in methylation between CT and KD. AluJb and AluSx showed consistently higher methylation in KD across all the methylation bins (top two panels), while AluY showed lower methylation in KD (bottom panel). (C) A subfamily classification of L1 repeats revealed that the L1HS and P family LINE1 such as L1P1, L1P2 and L1PA4 are reduced in KD although these repeat subtypes are more prevalent in highly methylated regions. (D) In contrast, the early originated LINE1 such as L1M1, L1M3, L1M4 and L1M5 showed increased methylation in KD and these repeat subtypes are prevalent in regions with low levels of methylation. [file 12863_2020_894_MOESM11_ESM.pdf]

A

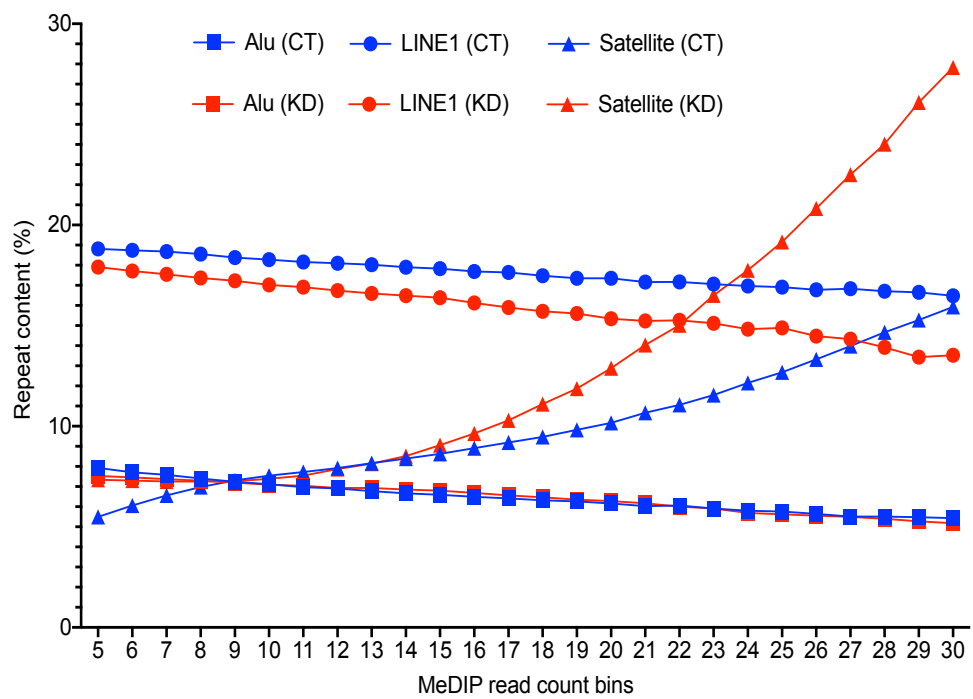

C

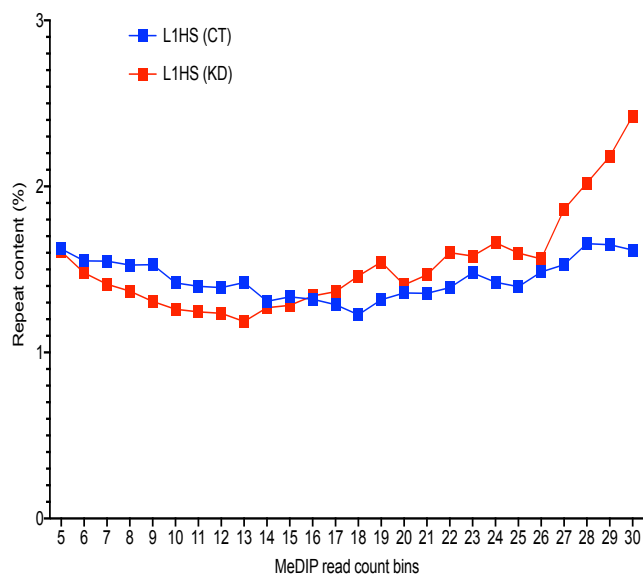

D

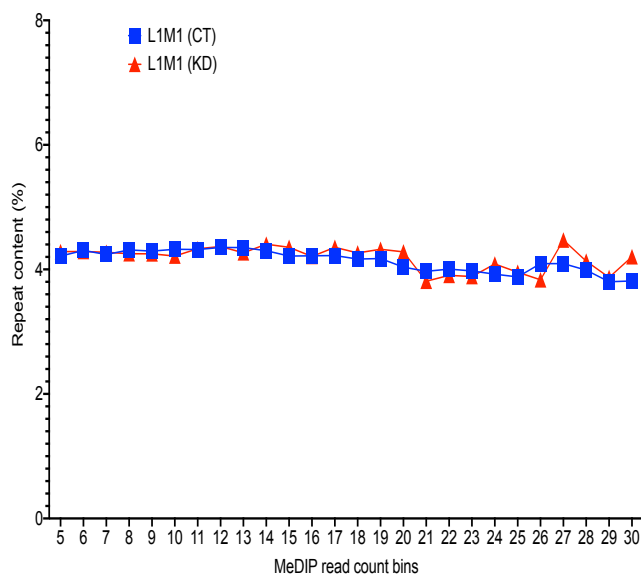

B

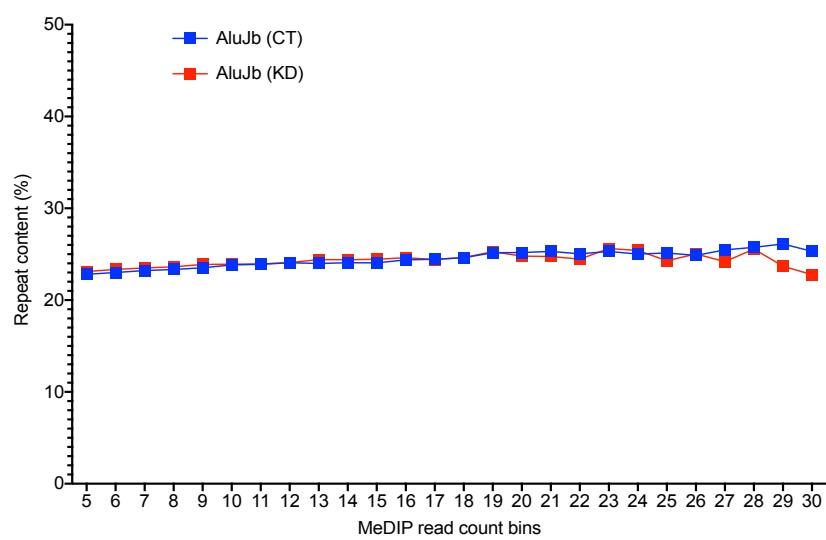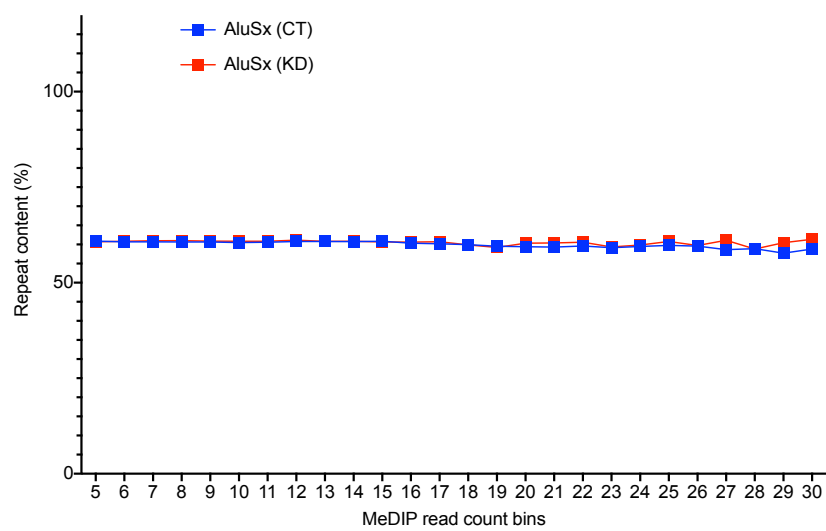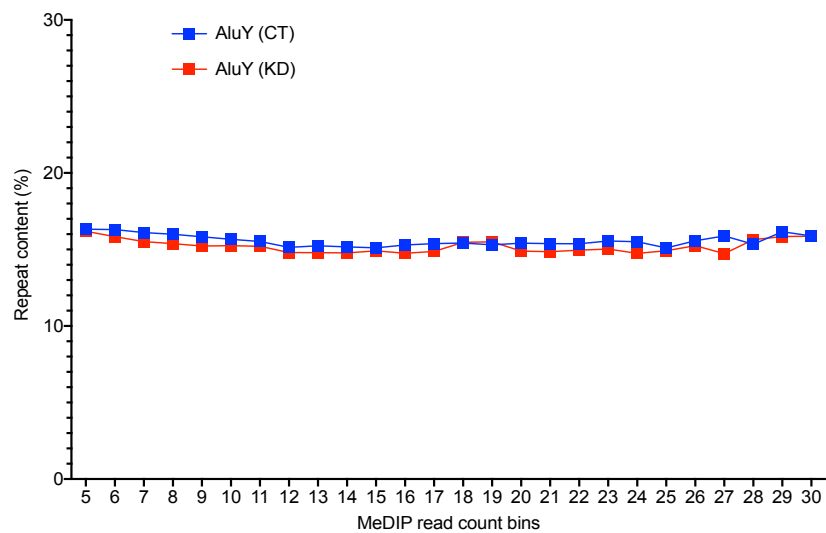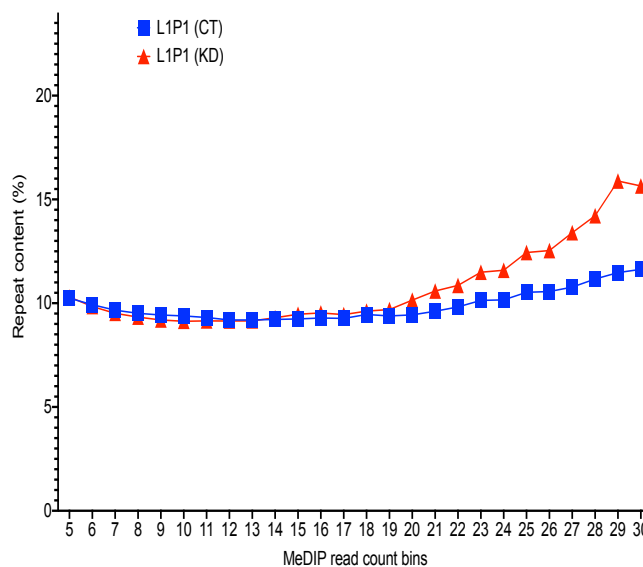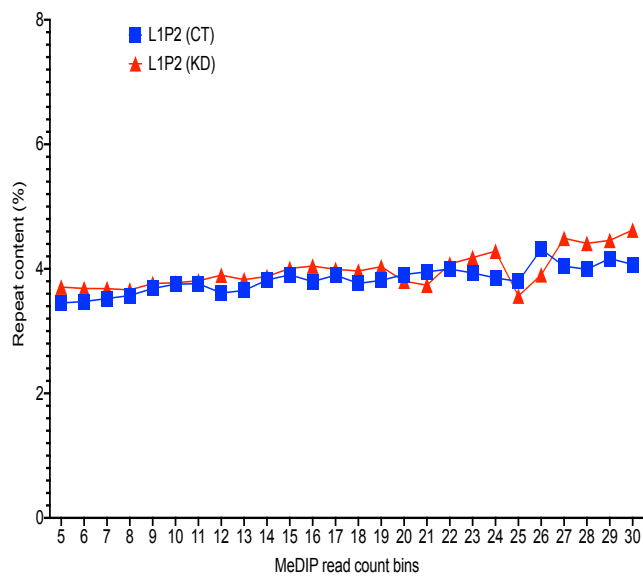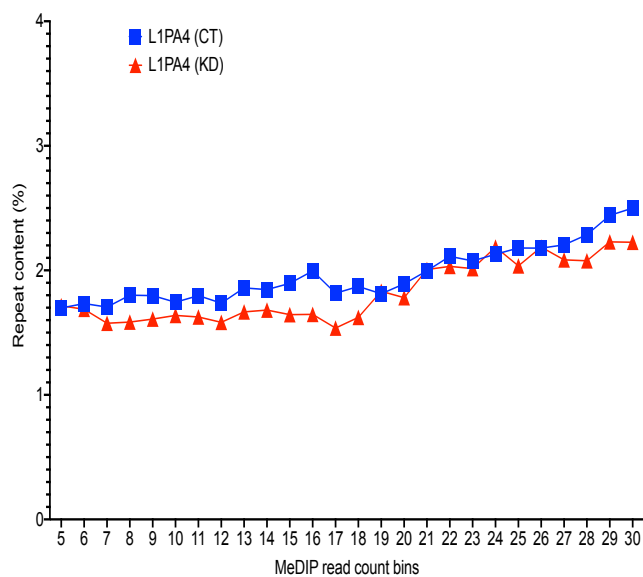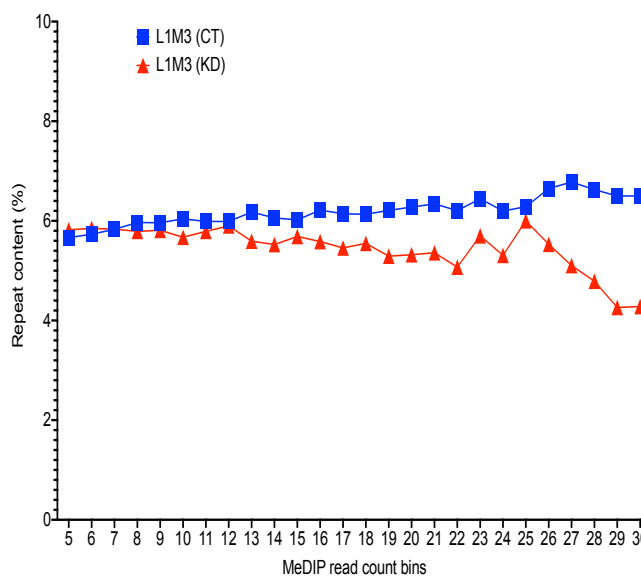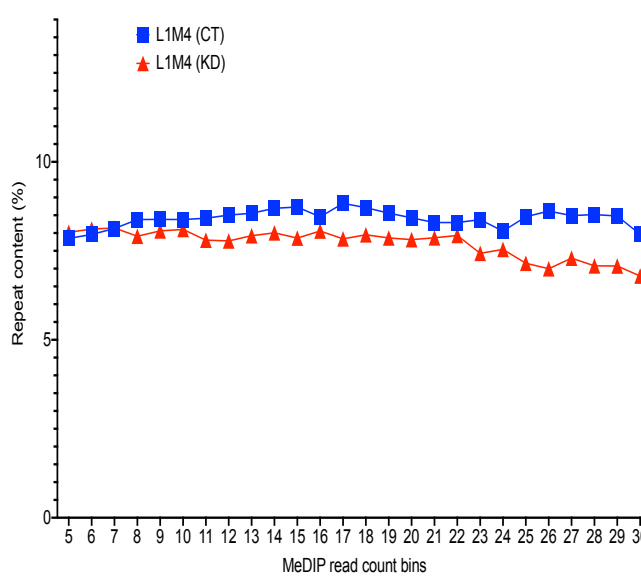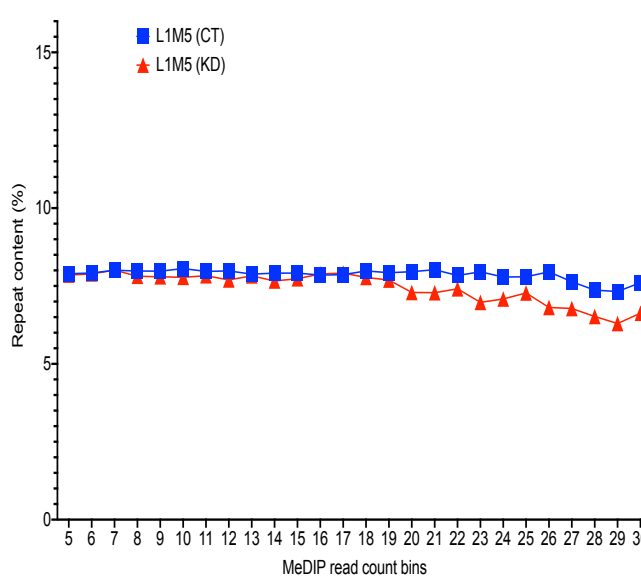

Supplement: Supplementary file 12 — Additional file 12. Repeat content analysis in GM02639 CT and KD MeDIP DNA shows subtle subfamily-specific methylation changes. CT and KD MeDIP repeat identification was performed as described for data in the Additional file 7. repeat identification and the occurrence of the three most populous repeats (Satellites, L1-LINEs and Alu-SINEs) were analyzed. (A) Satellite and L1 repeats were overrepresented and underrepresented respectively in KD. No difference in Alu-SINEs content was observed however. (B) Unlike HEK293T data, the subfamily classification of Alus does not reveal any subfamily-specific methylation differences between CT and KD in GM02639. (C) and (D): L1 repeat subfamily classification showed no consistent differences in methylation between CT and KD. [file 12863_2020_894_MOESM12_ESM.pdf]

CTCF  
(HoCoMoCo)

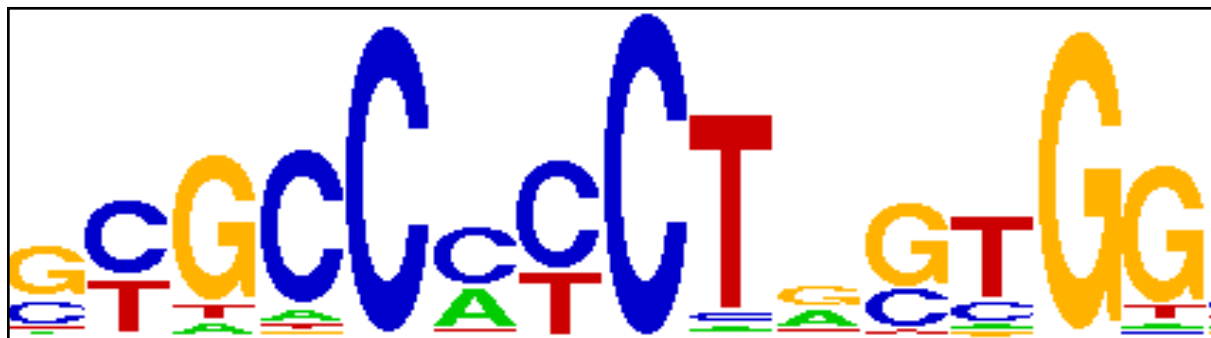

No Change

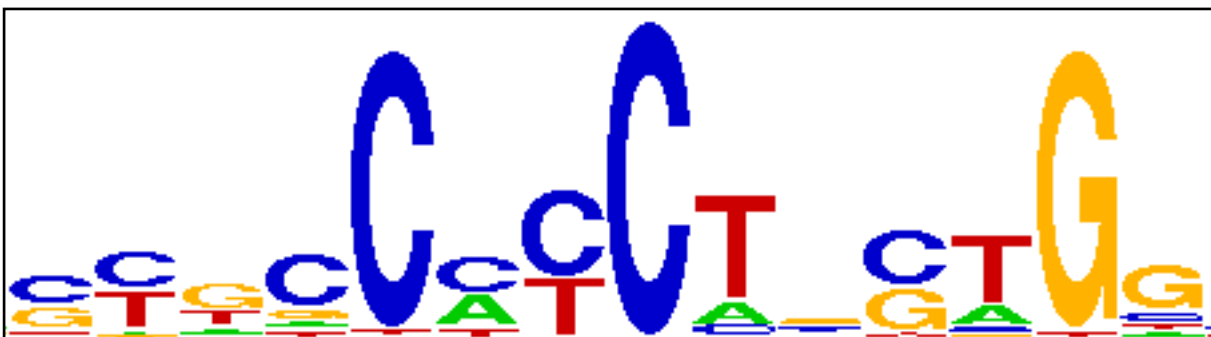

GoM

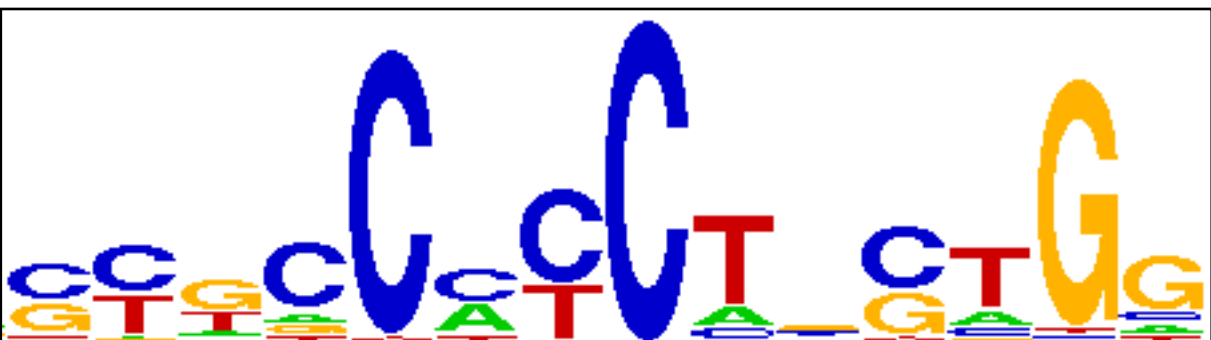

LoM

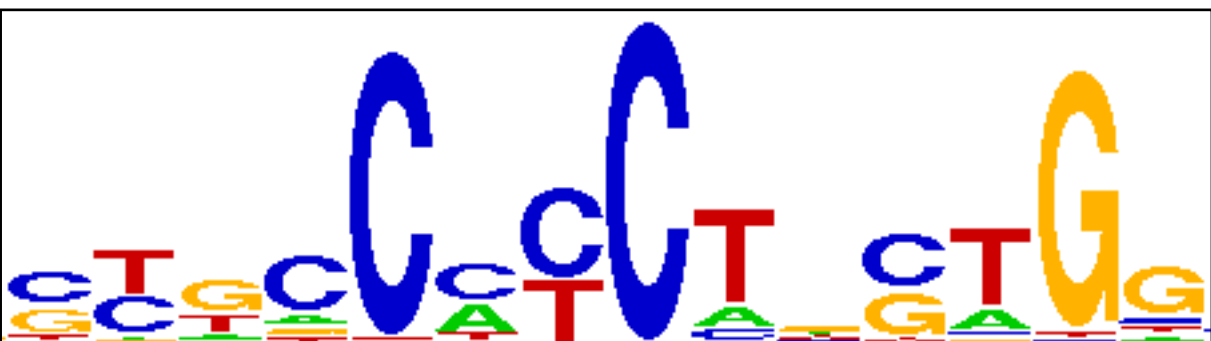

Supplement: Supplementary file 13 — Additional file 13. Highly similar CTCF binding motifs are present in regions undergoing GoM, LoM or showing no methylation change upon CGGBP1 depletion. Methylation signals for CT and KD were calculated for each 0.2 kb bin for HEK293T and bins were grouped into GoM, LoM and “No change” (as described in method in details). CTCF motif positive 0.2 kb bins were filtered out from each group. Motif positive GoM, LoM and “No change” 0.2 kb bin sequences were subjected to de novo motif search by using MEME suite. [file 12863_2020_894_MOESM13_ESM.pdf]

**A**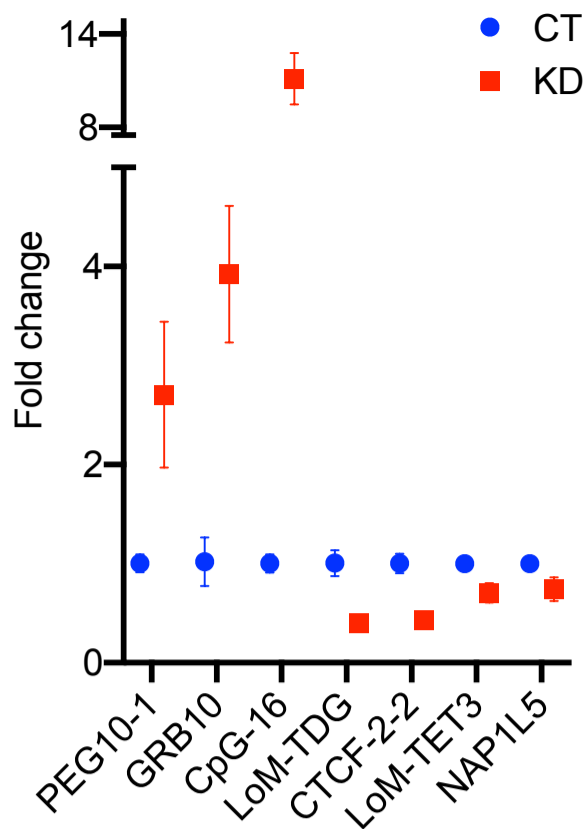**B**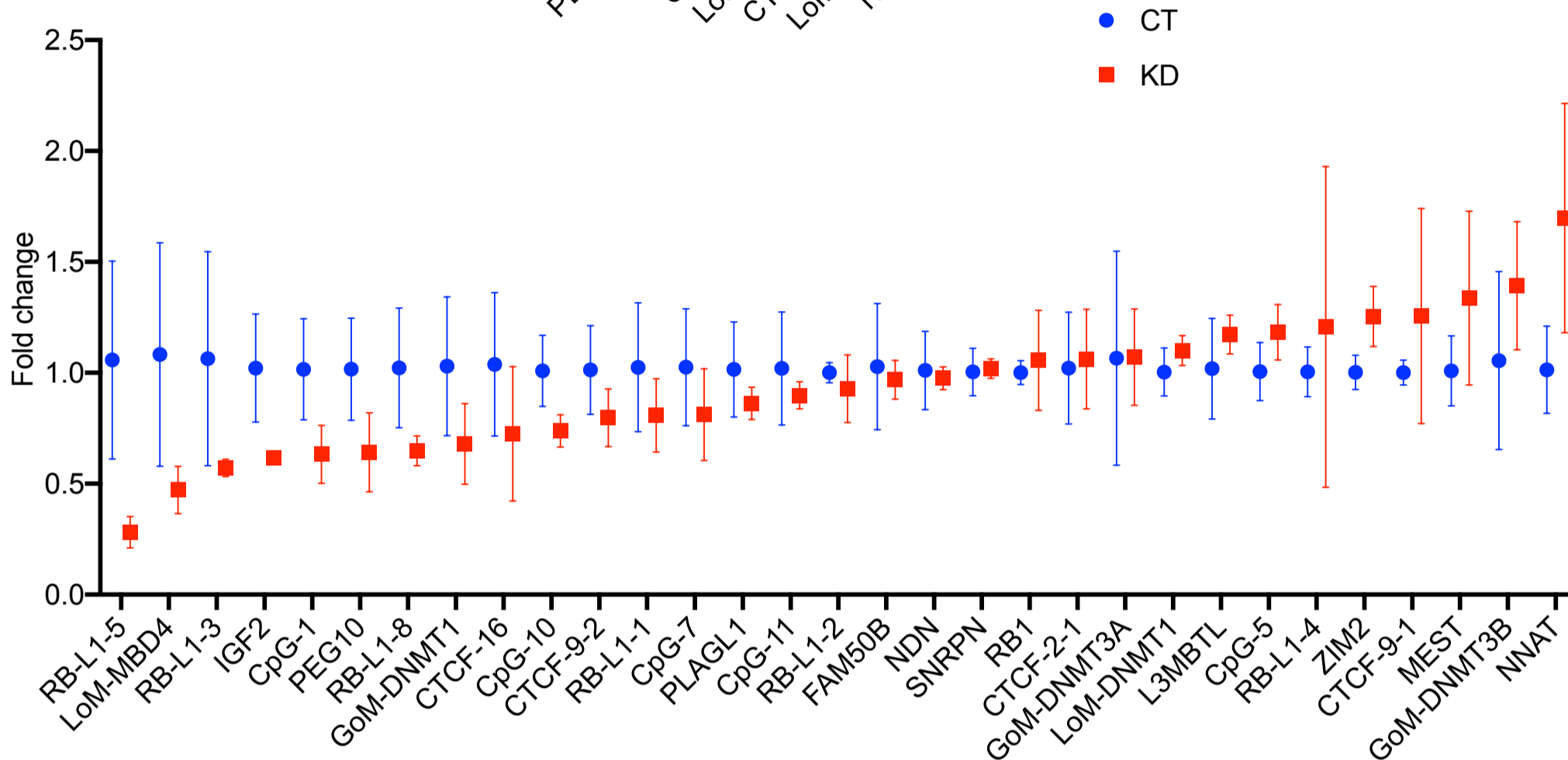**C**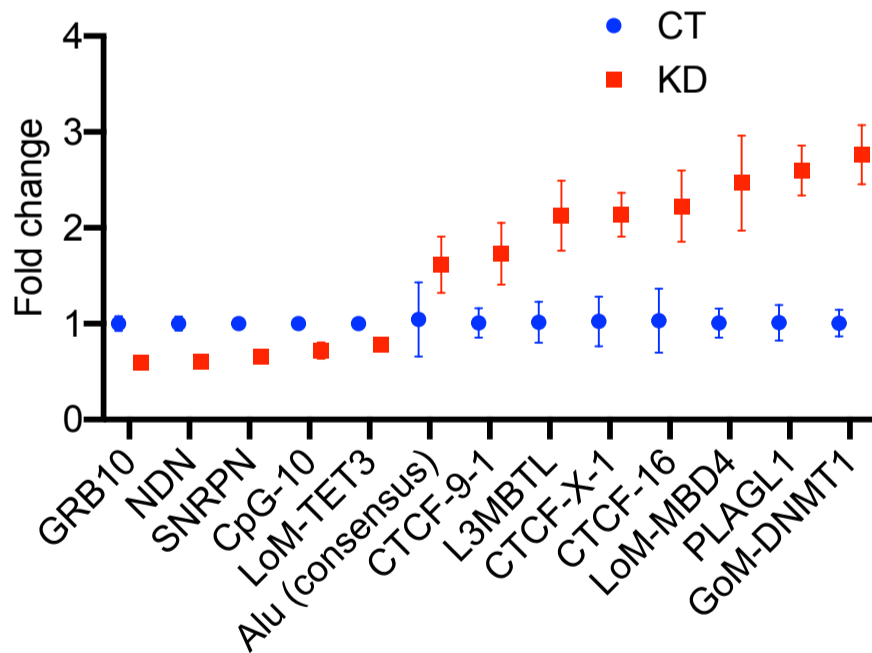**D**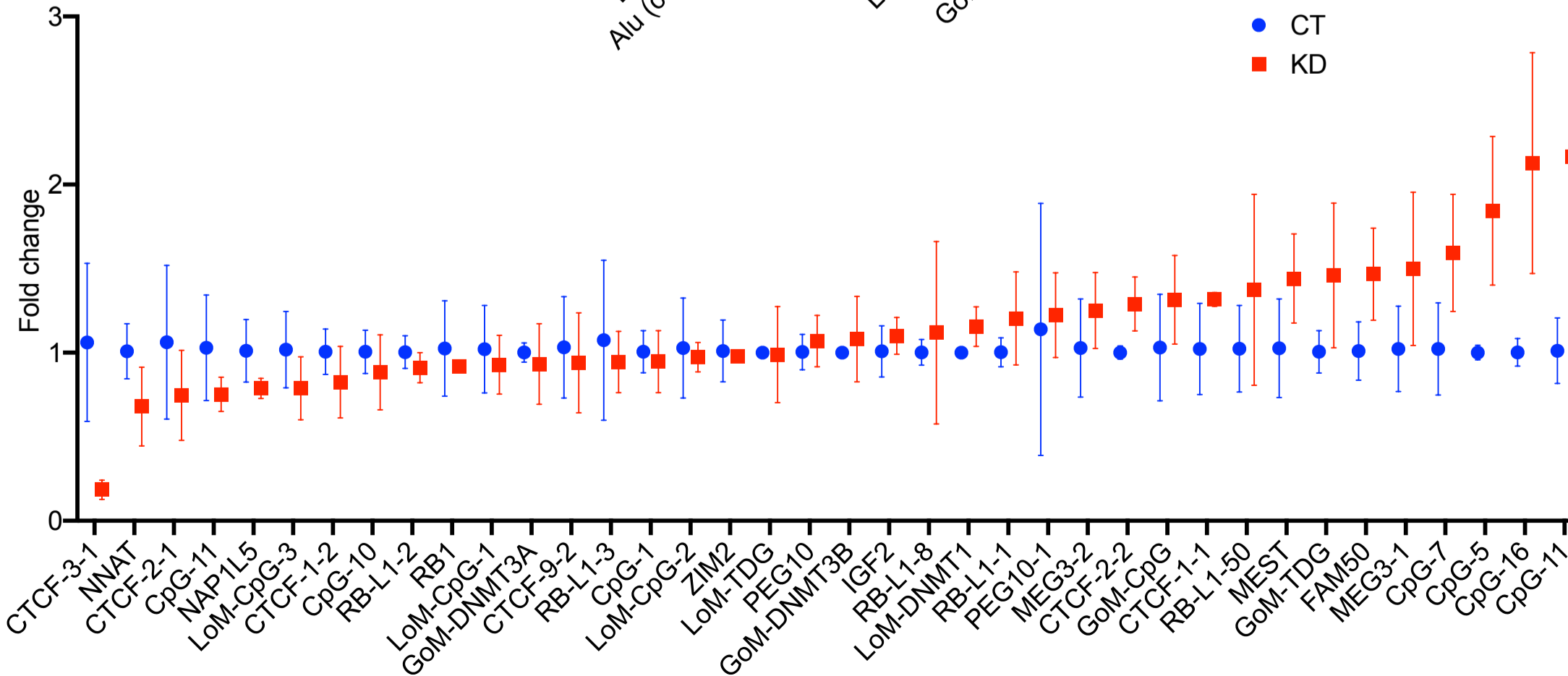

Supplement: Supplementary file 14 — Additional file 14 Quantitative PCR (double delta analysis of relative changes in levels of methylated DNA) on CT and KD DNA from human dermal fibroblasts shows widespread differences between CT and KD. (A) qPCR on HpaII-digested DNA (digests DNA flanking unmethylated cytosine) shows a significant (p < 0.05, n = 3 technical replicates, unpaired T test) gain of methylation at CpG sites for multiple genomic regions representing the PEG10 locus, GRB10 locus and a CpG island termed CpG-16. Conversely, a loci representing TDG, CTCF-binding site termed CTCF-2-2, TET3 and NAP1L5 showed a loss of methylation. All HpaII Ct values were normalized against corresponding Ct values obtained after MspI digestion. (B) Widespread CpG methylation disturbances (with no significance) were also observed at multiple other loci. (C) qPCRs on McrBC-digested CT and KD DNA showed a loss of methylation at non-CpG sites for multiple loci including Alu repeats and CTCF-binding sites (p < 0.05, n = 3 technical replicates, unpaired T test). (D) Several locations displayed widespread methylation disturbances (with no significance) as revealed by McrBC-digestion. Refer to Additional file 1 for exact location and PCR details. [file 12863_2020_894_MOESM14_ESM.pdf]
