# Supplementary material for: CGGBP1-regulated cytosine methylation at CTCF-binding motifs resists stochasticity
Source: BMC Genet. 2020 Jul 29;21:84. doi: 10.1186/s12863-020-00894-8 (PMC7392725; doi:10.1186/s12863-020-00894-8)
Supplement: Supplementary file 3 — Additional file 3. Tabulation of the sequencing and alignment statistics for CT and KD MeDIP in HEK293T and GM02639. [file 12863_2020_894_MOESM3_ESM.pdf]

Name	Total reads	Total mapped reads	Total unmapped reads	Reads % mapped	Reads % unmapped
HEK293T CT	75441659	70195180	5246479	93.046	6.954
HEK293T KD	76069183	69404104	6665079	91.238	8.762
GM02639 CT	118197258	94073184	24124074	79.59	20.41
GM02639 KD	83791601	45964460	37827141	54.856	45.144
GM02641	71104585	68684768	2419817	96.597	3.403
GM02640	96230084	92852408	3377676	96.49	3.51
