# Supplementary material for: CGGBP1-regulated cytosine methylation at CTCF-binding motifs resists stochasticity
Source: BMC Genet. 2020 Jul 29;21:84. doi: 10.1186/s12863-020-00894-8 (PMC7392725; doi:10.1186/s12863-020-00894-8)
Supplement: Supplementary file 4 — Additional file 4. Table represents GC content and percentage enrichment of CpG and non-CpG context cytosines in MeDIP read sequences for CT and KD in HEK293T and GM02639. The percentage of CpG, CHG and CHH represents the relative abundance for each cytosine context as a percentage of the total methylated cytosines enriched in MeDIP for CT and KD in HEK293T and GM02639. [file 12863_2020_894_MOESM4_ESM.pdf]

Samples	GC %	CpG %	CHG %	CHH %
HEK293T CT	42.21	5.42	21.51	73.07
HEK293T KD	42.49	5.67	21.64	72.69
GM02639 CT	40.47	7.80	21.00	71.20
GM02639 KD	40.87	13.45	21.12	65.44
