# Supplementary material for: CGGBP1-regulated cytosine methylation at CTCF-binding motifs resists stochasticity
Source: BMC Genet. 2020 Jul 29;21:84. doi: 10.1186/s12863-020-00894-8 (PMC7392725; doi:10.1186/s12863-020-00894-8)
Supplement: Supplementary file 6 — Additional file 6. The MeDIP signal correlation between CT and KD at different genomic bin sizes decline with a reduction in bin size specifically as randomization of coordinates (and thus corresponding sequences) changes the correlation stochastically away from the observed correlation coefficients with actual MeDIP sequences (refer to Additional file 5 for a comparison with correlation coefficients without any randomization). [file 12863_2020_894_MOESM6_ESM.pdf]

**10 Kb bins (Spearman r values)**							
CT::KD	0.92	CT shuffled::KD	0.38	KD shuffled::CT	0.39	CT shuffled:: KD shuffled	0.78
**5 Kb bins (Spearman r values)**							
CT::KD	0.87	CT shuffled::KD	0.35	KD shuffled::CT	0.36	CT shuffled:: KD shuffled	0.69
**0.2 Kb bins (Spearman r values)**							
CT::KD	0.51	CT shuffled::KD	0.15	KD shuffled::CT	0.15	CT shuffled:: KD shuffled	0.21
