# Supplementary material for: CGGBP1-regulated cytosine methylation at CTCF-binding motifs resists stochasticity
Source: BMC Genet. 2020 Jul 29;21:84. doi: 10.1186/s12863-020-00894-8 (PMC7392725; doi:10.1186/s12863-020-00894-8)
Supplement: Supplementary file 15 — Additional file 15. CpG SNPs in the entire AIM dataset and those mapping to the CTCF motifs undergoing AIM in GM02639 cells. The methylation changes at CTCF motifs are not linked to any detectable CpG SNPs within the motifs even if the flanking regions in the sequence reads harbour CpG SNPs. [file 12863_2020_894_MOESM15_ESM.pdf]

CpG SNP analysis summary of CTCF motifs showing AIM	
Total CTCF motifs (FIMO) showing AIM	8618
CTCF motifs with AIM containing 3 CpGs	11
CTCF motifs with AIM containing 2 CpGs	146
CTCF motifs with AIM containing 1 CpG	1701
CpG-devoid CTCF motifs with AIM	6760
Total number of Cytosine SNPs showing AIM	34318
CTCF motifs with AIM and non-CpG Cytosine SNPs	45
CTCF motifs with AIM and CpG Cytosine SNPs	0
Cytosine SNPs at sequence reads containing the CTCF motifs with AIM	1153
